# Computerized Diagnostic Assistant for the Automatic Detection of Pneumothorax on Ultrasound: A Pilot Study

**DOI:** 10.5811/westjem.2016.1.28087

**Published:** 2016-03-02

**Authors:** Shane M. Summers, Eric J. Chin, Brit J. Long, Ronald D. Grisell, John G. Knight, Kurt W. Grathwohl, John L. Ritter, Jeffrey D. Morgan, Jose Salinas, Lorne H. Blackbourne

**Affiliations:** * Brooke Army Medical Center, Department of Emergency Medicine, San Antonio, Texas; † Brooke Army Medical Center, Department of Radiology, San Antonio, Texas; ‡ Brooke Army Medical Center, Department of Pulmonary/Critical Care, San Antonio, Texas; § United States Army Institute of Surgical Research, San Antonio, Texas; ¶ United States Army Medical Department Center and School

## Abstract

**Introduction:**

Bedside thoracic ultrasound (US) can rapidly diagnose pneumothorax (PTX) with improved accuracy over the physical examination and without the need for chest radiography (CXR); however, US is highly operator dependent. A computerized diagnostic assistant was developed by the United States Army Institute of Surgical Research to detect PTX on standard thoracic US images. This computer algorithm is designed to automatically detect sonographic signs of PTX by systematically analyzing B-mode US video clips for pleural sliding and M-mode still images for the seashore sign. This was a pilot study to estimate the diagnostic accuracy of the PTX detection computer algorithm when compared to an expert panel of US trained physicians.

**Methods:**

This was a retrospective study using archived thoracic US obtained on adult patients presenting to the emergency department (ED) between 5/23/2011 and 8/6/2014. Emergency medicine residents, fellows, attending physicians, physician assistants, and medical students performed the US examinations and stored the images in the picture archive and communications system (PACS). The PACS was queried for all ED bedside US examinations with reported positive PTX during the study period along with a random sample of negatives. The computer algorithm then interpreted the images, and we compared the results to an independent, blinded expert panel of three physicians, each with experience reviewing over 10,000 US examinations.

**Results:**

Query of the PACS system revealed 146 bedside thoracic US examinations for analysis. Thirteen examinations were indeterminate and were excluded. There were 79 true negatives, 33 true positives, 9 false negatives, and 12 false positives. The test characteristics of the algorithm when compared to the expert panel were sensitivity 79% (95 % CI [63–89]) and specificity 87% (95% CI [77–93]). For the 20 images scored as highest quality by the expert panel, the algorithm demonstrated 100% sensitivity (95% CI [56–100]) and 92% specificity (95% CI [62–100]).

**Conclusion:**

This novel computer algorithm has potential to aid clinicians with the identification of the sonographic signs of PTX in the absence of expert physician sonographers. Further refinement and training of the algorithm is still needed, along with prospective validation, before it can be utilized in clinical practice.

## INTRODUCTION

In the hands of appropriately trained clinicians, bedside thoracic ultrasound (US) can rapidly diagnose pneumothorax (PTX) with improved accuracy over the physical examination and without the need for chest radiography (CXR). In a recent meta-analysis, the pooled sensitivity and specificity of bedside thoracic US to detect PTX was 90.9% and 98.2%, respectively.^1^ However, the major hurdle in realizing the full potential of bedside thoracic US is the implementation of an effective training program that ensures the competency of the sonographer, and at least one prior study demonstrated that operator skill is highly correlated with diagnostic accuracy.^2^ To aid the US novice in the absence of expert clinical sonographers, such as in austere environments or community settings, a computerized diagnostic assistant was created by biomedical software engineers at the United States Army Institute of Surgical Research (USAISR). This computer algorithm systematically analyzes B-mode US video clips for pleural sliding as well as M-mode still images for the seashore sign, which are indicators of normal aerated lung.^1^

Computer programs that assist clinicians in the interpretation of diagnostic studies are not a novel concept.^3^–^18^ For example, electrocardiogram (EKG)-reading software is in widespread use today and can assist non-cardiologists in achieving more uniform and consistent interpretations.^18^ To our knowledge, there has been only one publication examining the potential of an automated thoracic US interpretation system.^19^ In this study, a computer algorithm to diagnose acute pulmonary edema on thoracic US was described. Although our concept is similar to the aforementioned study, we believe that there is no published research specifically evaluating the potential for an automated system to detect PTX.

The primary objective was to obtain a baseline estimate of the diagnostic accuracy of our computerized diagnostic assistant to detect sonographic signs of PTX. The secondary objectives were to analyze its performance after stratifying the examinations by image quality, mode of imaging, and transducer selection in order to guide future prototype development.

## METHODS

### Study Design

We conducted a retrospective pilot study using bedside thoracic US images obtained on adult emergency department (ED) patients between May 23, 2011, and August 6,2014. We sampled images from the Picture Archive and Communications System (PACS) through query of our US quality assurance database (Filemaker Pro, Santa Clara, CA). The computerized diagnostic assistant analyzed images and reported the result as positive, negative, or indeterminate for PTX. A blinded, independent expert panel of three physician sonographers then reviewed the same images and recorded their interpretations on a standardized data collection sheet. For the primary outcome, we compared the algorithm’s interpretation to the consensus decision of the expert panel. The study was approved by the institutional review board with a waiver of informed consent.

### Study Setting

**Figure 1:**
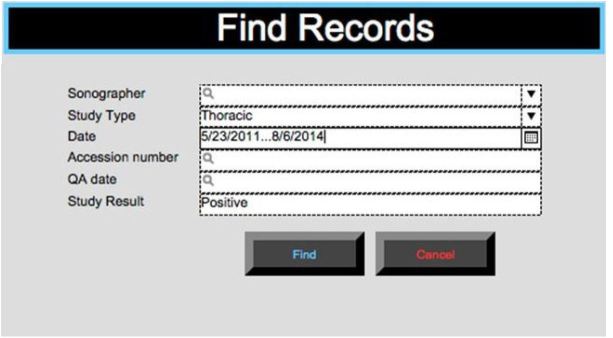
Example of the thoracic ultrasound reporting template in the emergency department quality assurance database (Filemaker Pro, Santa Clara, CA).

The study setting was an academic level I trauma center ED with an annual census of 77,000 patients. All US images were acquired with the Sonosite M-turbo or S-FAST system (Bothell, WA), based on clinician preference. At our institution, we support a robust training and quality assurance (QA) program for ED US and perform approximately 7,000 bedside examinations per year. Our QA process entails a weekly review of 100% of archived ED bedside US examinations. During these QA sessions, faculty input a report for each study into a Filemaker Pro (Santa Clara, CA) computer database (Figure 1). This report contains the study type and date, the sonographer, the study result, and a unique 8-digit accession number that links to the images in PACS.

### Study Protocol

We queried the Filemaker Pro (Santa Clara, CA) database for all positive bedside thoracic US examinations reported during the study period on ED patients aged 18–89 years. We then inputted the accession numbers into the PACS and exported the images to a compact disc (CD). After the positives were collected, we queried the database for a random sample of negative thoracic US examinations obtained during the same study period. For convenience, we chose to sample a 2:1 ratio of negative examinations to positives to maximize efficiency. A 1:1 ratio would require over 1,000 negative studies. In order to collect the negatives, we used http://www.randomization.com to generate a single column of random two-digit integers, and we performed a database search for US studies with accession numbers ending in these two digits. We continued searching the database with each generated two-digit number down the column until we had achieved a 2:1 ratio. The negative PTX images were then exported from the PACS in the same fashion as the positives. Finally, all of the positive and negative images were de-identified and placed in random order on four identical CDs, one for each expert panel member and one for the computer algorithm.

### Measurements

#### The Computerized Diagnostic Assistant

Biomedical software engineers at the USAISR, in consultation with emergency medicine US fellowship-trained physicians, developed the PTX computerized diagnostic assistant as the first phase of the intelligent focused assessment with sonography for trauma (iFAST) project.^20^ The patent application for this project was published on April 2, 2015. 20 The PTX algorithm was initially trained on a de-identified set of 80 positive and 80 negative thoracic US images used by the fellowship for teaching purposes.

**Figure 2:**
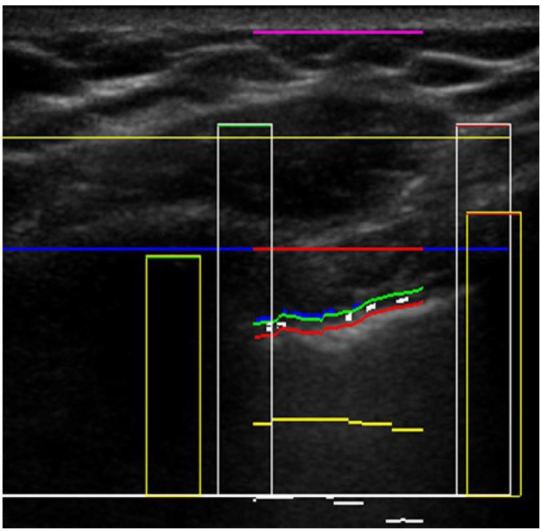
In this B-mode image, the intelligent focused assessment with sonography for trauma (iFAST) has correctly identified the pleural line in order to examine for sliding lung sign. The purple line denotes the skin surface. The first horizontal yellow line is the pectoralis muscle. To find the pleural line, the iFAST first locates the rib shadows (yellow rectangles). The red break in the blue horizontal line between the ribs defines the intercostal space. The pleural line appears like a road with paired green and red horizontal lines in the intercostal space. The small white rectangles on the “road” denote pixel movements back and forth along the pleural line, indicating normal sliding lung.

For thoracic B-mode images, the iFAST is designed to detect the presence of the sliding lung sign, which is indicative of normal apposition of the visceral and parietal surfaces.^21^ It is also capable of identifying common reverberation artifacts that can assist with the identification of PTX in standard B-mode imaging.^22^ In the first step of the B-mode algorithm, two discrete ribs with posterior acoustic shadowing assist the device in locating the intercostal space on the US image (Figure 2). The iFAST identifies the pleural line by searching for a hyper-echoic linear and contiguous line that runs immediately beneath the ribs in the intercostal space. While focusing on the pleural line, it dynamically scans each frame of the respiratory cycle, searching for horizontal pixel movements to and fro in a coordinated fashion as well as reverberation artifacts. If the algorithm identifies pixel movement back and forth along the pleural line or pleural line reverberations, it will report negative for PTX. If pixel movement cannot be detected and there are no reverberation artifacts extending below, then it will report positive for PTX. If it cannot identify the pleural line at all, it will report as indeterminate.

The iFAST was also trained to analyze M-mode US images of the thorax. In normal M-mode imaging, horizontal movement along the pleural line will cause granular or speckled artifacts to appear below the pleural line resembling a sandy beach, also known as the seashore sign.^21^ With PTX, absence of motion due to interposed air will create a barcode pattern with linear artifacts below the pleural line, known as the stratosphere sign.^21^ The M-mode algorithm first identifies the pleural line as the most hyper-echoic contiguous line on the screen, and then it analyzes below for granularity or barcode pattern.

#### The Expert Panel

The primary outcome was the consensus decision of an independent expert panel of physician sonographers. This panel consisted of one board-certified radiologist (JR), one board-certified pulmonary/critical care physician with Registered Diagnostic Medical Sonographer certification (KG), and one US fellowship-trained emergency physician (JK), each with experience reviewing over 10,000 US examinations. The expert panel members were blinded to all clinical data as well as to the iFAST interpretation. No expert panel member was involved in the iFAST project or patenting of the computer algorithm.

Each panel member received a CD along with a separate standardized data collection instrument. The panel members individually reviewed the images and recorded an interpretation along with an image quality score. The scoring system used was a five-point Likert scale (Table 1) recommended for US quality assurance by the 2011 American College of Emergency Physicians Ultrasound Standard Reporting Guidelines.^23^ By convention, images with a score of three or greater yielded diagnostic information, whereas scores of one or two were deemed indeterminate.

After the expert panel members completed their independent review of the US images, we examined their interpretations for concordance. If all panel members unanimously agreed on the study result, then the US images were included in the study. For the studies in which there was initial disagreement, the expert panel had a plenary discussion to determine consensus. If the panel could not reach a consensus, then the study was excluded. Thirteen scans were excluded, resulting in analysis of 133 scans. Indeterminate scans with scores below three on the American College of Emergency Physiciancs (ACEP) image quality scale were excluded from study. This scale is shown in Table 1.

## ANALYSIS

For the primary analysis, we compared the iFAST interpretation to the expert panel consensus decision to determine test characteristics along with the corresponding 95% confidence intervals. Cohen’s Kappa was calculated to determine initial agreement between the three expert panel members. A Kappa from 0.40 to 0.75 indicated fair to good agreement. A Kappa >0.75 indicated excellent agreement.

For the secondary analysis, we stratified the US examinations by image quality scores and analyzed the performance of the algorithm in these subgroups. In order to hypothesize about proper mode and transducer selection for future prospective studies, we created contingency tables for the test performance for both B-mode versus M-mode images as well as linear versus phased array probes.

## RESULTS

**Figure 3:**
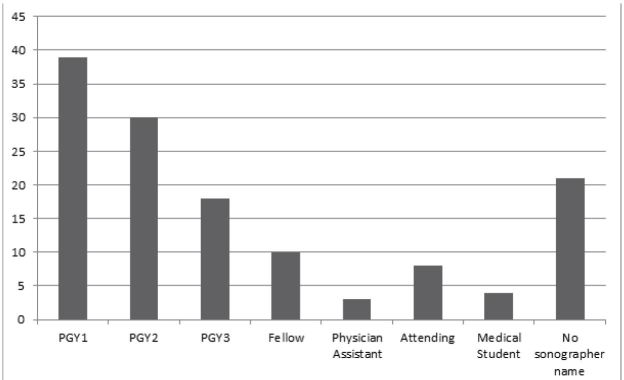
Post-graduate year (PGY) level of the sonographers who performed the bedside thoracic ultrasound (US) examinations. Twenty-one sonographers failed to input their name on the US study at the time of imaging; thus, their PGY level could not be ascertained.

**Figure 4:**
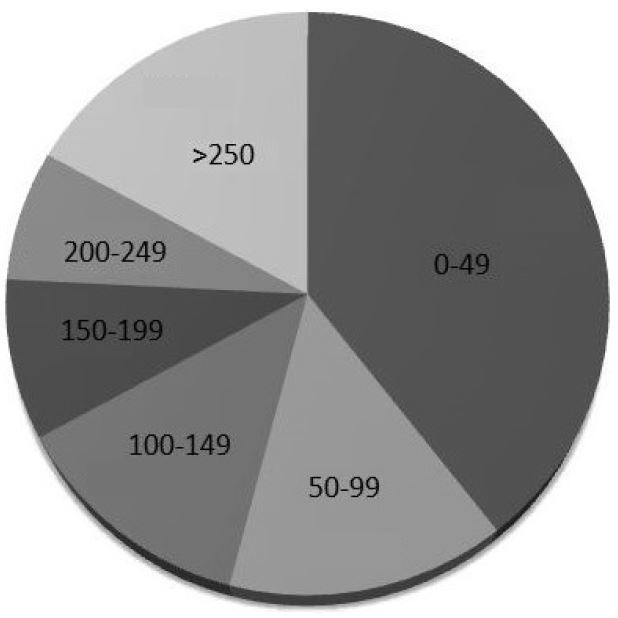
Prior ultrasound experience of the sonographers who performed the bedside thoracic ultrasound examinations.

Query of the Filemaker Pro (Santa Clara, CA) QA database revealed 49 bedside thoracic US examinations reported as positive for PTX along with 98 randomly sampled negative examinations. The examinations were performed by sonographers from all post-graduate year (PGY) training levels (Figure 3) with a wide range of prior US experience (Figure 4). After excluding one study for patient age, 146 images were exported from the PACS and copied onto a CD for review by the expert panel and the iFAST. Thirteen of these images were reported as indeterminate and were excluded from final data analysis, leaving 133 scans for analysis. When the iFAST interpretation was compared to the expert panel as the gold standard, there were 79 true negatives, 33 true positives, 9 false negatives, and 12 false positives. For the primary outcome, the overall test characteristics of the algorithm were as follows: sensitivity 79% (95 % CI [63–89]), specificity 87% (95% CI [77–93]).

Our results demonstrated excellent agreement for the expert panel. After the first independent blinded review, there was unanimous agreement between the three expert panel members for 90% of US examinations. Consensus decision on the final result was reached for all of the remaining studies during the plenary discussion. Initial agreement between the first expert panel reviewer (KG) and the second reviewer (JK) was Kappa 0.84 (95% CI [0.75–0.93]), between the first (KG) and third reviewer (JR) was Kappa 0.86 (95% CI [0.78–0.95]), and between the second (JK) and third (JR) was Kappa 0.83 (0.74–0.92).

For the secondary analysis, the range of image quality scores was from 2 to 5, (mean 3.8, median 4; interquartile range 3 to 4). The iFAST performed well when interpreting US examinations with an image quality score of 5, although the sample size was too small to draw definite conclusions (Table 2). Table 3 reports the test characteristics of the iFAST when grouped by mode of imaging and transducer selection. The iFAST appeared to perform with higher sensitivity for B-mode images and with the phased array transducer, although there were no statistically significant differences.

## DISCUSSION

The iFAST algorithm was initially developed by the USAISR as a potential diagnostic tool to assist combat medics in austere environments. Thoracic US in the prehospital setting may alter management for injured patients, such as the evacuation destination, the evacuation platform, or the need for tube thoracostomy.^24^ However, in one study of thoracic US performed by aeromedical transport teams, the sensitivity for detection of PTX was only 18.7%.^25^ Furthermore, training prehospital providers across a wide variety of systems presents significant logistical challenges such as ensuring skill retention and providing quality assurance. The iFAST was designed to mitigate these challenges by providing novice sonographers with a reliable computerized diagnostic assistant capable of recognizing common sonographic signs of PTX. The iFAST could be useful in a prehospital or aeromedical environment where conditions are not optimal and novice sonographers may be present, but further refinement and study of the algorithm is required for these settings. The purpose of this pilot study was to estimate the diagnostic accuracy of the algorithm, provide proof of concept, and determine needs for future prototype development.

In our pilot study, the iFAST was 79% sensitive and 87% specific, which we believe supports proof of concept and is encouraging considering the non-standardized way in which the images were recorded. For the images with quality scores of five, the iFAST performed well, which suggests that the actual computer algorithm can work if it “sees” a good image. However, our overall results were far from optimal and cannot be used to support the current clinical use of the iFAST over the experience of US-trained personnel.

To improve image interpretation capabilities, we plan to train the iFAST with a larger sample of known positive and negative thoracic US images. However, the bigger challenge will be to standardize the image acquisition process in such a way that the iFAST can be given the best chance to render an accurate interpretation. Much like with EKG computerized programs where a reliable reading depends on the correct location of electrodes, an accurate algorithm in clinical practice will require correct placement of the transducer.^18^ Because of retrospective design, we do not know if controlling the methods in which the images were obtained would have improved the diagnostic accuracy. However, based on the performance of the algorithm with image quality scores of five, we believe that our primary focus for future prototype development should be standardizing image acquisition rather than aggressive retraining of the computer software. Because our device will ultimately be intended for novices, the guidelines for image acquisition should be relatively straightforward and easily employed. The logical next step to assess the iFAST should be a prospective evaluation of US novices using the algorithm in a blinded, predefined fashion and comparing the results to computed tomography (CT).

## LIMITATIONS

This was a small retrospective study at a single center with a robust US training program; thus, our results may not be generalizable. The retrospective nature of this study precluded controlling certain parameters such as depth, frequency, and mode, and there may have been image quality degradations when exporting US video secondary to data compression. Also, sonographers may have chosen not to archive examinations if there was technical difficulty, leading to potential selection bias. Using a convenience sample of negatives may also have caused selection bias; however, we did randomly select the negatives to improve our chances of achieving a representative sample. By using a 2:1 ratio for negative to positive findings, investigators controlled prevalence, thus affecting the positive and negative predictive values. Using a 2:1 ratio is indeed a limitation.

Another major limitation of our study was the choice of the expert panel as the gold standard rather than CT. We chose these methods in order to hypothesize whether the predominant source of iFAST diagnostic error was due to image quality, transducer placement, or an inherent flaw in the software’s interpretation of the images. For example, if the algorithm missed the sonographic signs of PTX when compared to experts, especially with a high-quality image, this would suggest a flaw in the interpretation algorithm and would require retraining. If the iFAST correctly interpreted the presence of sliding lung, multiple US experts agreed that the image was negative, but the CT was positive, this could suggest that the transducer was not placed in the correct intercostal space to “see” the PTX. The latter problem, while still important, would not necessarily mandate retraining of the computer. Rather, it would require solutions to assist the novice operator obtain reliable images for the software to interpret. Ultimately, controlling image acquisition and ensuring reliable software interpretation will be critical for successful deployment of the iFAST to novices in a clinical setting; however, each requires a different focus for future prototype development.

## CONCLUSION

In the absence of expert physician sonographers, the iFAST computerized diagnostic assistant has potential to aid clinicians with the identification of the sonographic signs of PTX. This algorithm has additional potential in settings in which conditions are not optimal for US use and novice sonographers are present. However, in its current form, the iFAST is not sufficiently sensitive or specific for lower quality US images when compared with expert physician interpretation. The optimal image acquisition process to ensure reliable readings must be further defined, standardized, and validated in future prospective studies before it can be deployed in clinical practice.

## Figures and Tables

**Table 1:** American College of Emergency Physiciancs (ACEP) emergency ultrasound standard reporting guidelines.

	1	2	3	4	5
Grading scale definitions	No recognizable structures, no objective data can be gathered	Minimally recognizable structures but insufficient for diagnosis	Minimal criteria met for diagnosis, recognizable structures but with some technical or other flaws	Minimal criteria met for diagnosis, all structures imaged well and diagnosis easily supported	Minimal criteria met for diagnosis, all structures imaged with excellent image quality and diagnosis completely supported

**Table 2:** Overall test characteristics of the intelligent focused assessment with sonography for trauma (iFAST) when compared to the expert panel interpretation with results stratified by image quality score.

Test characteristics	Overall N=133 (95% CI)	Image quality 3 N=45 (95% CI)	Image quality 4 N=68 (95% CI)	Image quality 5 N=20 (95% CI)
Sensitivity, %	79 (63–89)	73 (39–93)	75 (53–89)	100 (65–100)
Specificity, %	87 (78–93)	88 (72–96)	84 (69–93)	92 (62–100)
PPV, %	73 (58–85)	67 (35–88)	72 (50–87)	88 (47–99)
NPV, %	90 (81–95)	91 (75–98)	86 (71–94)	100 (70–100)

*PPV,* positive predictive value; *NPV,* negative predictive value

**Table 3:** Test characteristics of the intelligent focused assessment with sonography for trauma (iFAST) when stratified by mode of imaging and transducer selection.

Test characteristics	B-mode N=107 (95% CI)	M-mode N=26 (95% CI)	Linear array N=80 (95% CI)	Phased array N=53 (95% CI)
Sensitivity, %	87 (68–96)	58 (29–84)	76 (57–88)	89 (51–99)
Specificity, %	86 (75–92)	93 (64–100)	87 (74–95)	86 (72–94)
PPV, %	65 (56–74)	88 (47–99)	81 (62–92)	57 (30–81)
NPV, %	94 (85–98)	72 (46–89)	84 (70–92)	97 (85–100)

*PPV,* positive predictive value; *NPV,* negative predictive value
